# Anti-Inflammatory and Osteogenic Effect of Phloroglucinol-Enriched Whey Protein Isolate Fibrillar Coating on Ti-6Al-4V Alloy

**DOI:** 10.3390/polym17111514

**Published:** 2025-05-29

**Authors:** Anna Mieszkowska, Laurine Martocq, Andrey Koptyug, Maria A. Surmeneva, Roman A. Surmenev, Javad Naderi, Maria Muchova, Katarzyna A. Gurzawska-Comis, Timothy E. L. Douglas

**Affiliations:** 1 Department of Evolutionary Immunology, Institute of Zoology and Biomedical Research, Faculty of Biology, Jagiellonian University, 30-387 Krakow, Poland; 2 School of Engineering, Lancaster University, Lancaster LA1 4YW, UK; laurine.martocq@gmail.com (L.M.); t.douglas@lancaster.ac.uk (T.E.L.D.); 3 Department of Quality Technology, Mechanical Engineering & Mathematics, Mid Sweden University, 851 70 Östersund, Sweden; andrey.koptyug@miun.se; 4 Physical Materials Science and Composite Materials Centre, Research School of Chemistry & Applied Biomedical Sciences, National Research Tomsk Polytechnic University, 634050 Tomsk, Russia; surmenevamaria@mail.ru (M.A.S.); rsurmenev@mail.ru (R.A.S.); 5 Chemistry Department, Lancaster University, Lancaster LA1 4YW, UK; javadnaderi107@gmail.com; 6 Institute for Medical Microbiology and Virology, Leipzig University, 04103 Leipzig, Germany; maria.muchova@medizin.uni-leipzig.de; 7 Periodontal Research Group, Birmingham School of Dentistry, Institute of Clinical Sciences, The University of Birmingham, Birmingham B15 2TT, UK; 8 Department of Oral Surgery, Liverpool University Dental Hospital, Institute of Life Course and Medical Science, University of Liverpool, Liverpool L3 5PS, UK; k.gurzawska-comis@liverpool.ac.uk; 9 Department Musculoskeletal Ageing Science, Institute of Life Course and Medical Science, University of Liverpool, Liverpool L3 5PS, UK; 10 Liverpool Head and Neck Centre, University of Liverpool, Liverpool L69 3BX, UK; 11 Department of Dentistry and Oral Health, Section for Maxillofacial Surgery and Oral Pathology, Aarhus University, Vennelyst Boulevard 9, DK-8000 Aarhus, Denmark

**Keywords:** Ti6Al4V, fibrillar coating, phenolic coating, whey protein isolate, inflammation, biofilm, osseointegration, osteogenesis, stem cells

## Abstract

Biomaterials play a crucial role in the long-term success of bone implant treatment. The accumulation of bacterial biofilm on the implants induces inflammation, leading to implant failure. Modification of the implant surface with bioactive molecules is one of the strategies to improve biomaterial compatibility and limit inflammation. In this study, whey protein isolate (WPI) fibrillar coatings were used as a matrix to incorporate biologically active phenolic compound phloroglucinol (PG) at different concentrations (0.1% and 0.5%) on titanium alloy (Ti6Al4V) scaffolds. Successful Ti6Al4V coatings were validated by X-ray photoelectron spectroscopy (XPS), showing a decrease in %Ti and increases in %C, %N, and %O, which demonstrate the presence of the protein layer. The biological activity of PG-enriched WPI (WPI/PG) coatings was assessed using bone-forming cells, human bone marrow-derived mesenchymal stem cells (BM-MSCs). WPI/PG coatings modulated the behavior of BM-MSCs but did not have a negative impact on cell viability. A WPI with higher concentrations of PG increased gene expression relative to osteogenesis and reduced the pro-inflammatory response of BM-MSCs after biofilm stimulation. Autoclaving reduced WPI/PG bioactivity compared to filtration. By using WPI/PG coatings, this study addresses the challenge of improving osteogenic potential while limiting biofilm-induced inflammation at the Ti6Al4V surface. These coatings represent a promising strategy to enhance implant bioactivity.

## 1. Introduction

Titanium (Ti) and its alloys, in particular Ti6Al4V, are widely used as primary biomaterials for orthopedic and dental implants due to their good physical–chemical properties and high biocompatibility with host tissues. However, despite these advantages, the implantation of Ti into bone disrupts host tissues and induces an inflammatory response. Immune cells and mesenchymal stem cells (MSCs) that are recruited to the site of implantation play key roles in the bone-healing process. MSCs differentiate into mature bone-forming cells and deposit a collagen matrix, leading to bone formation at the implant surface [1,2]. This complex and long-term process could be affected by different factors, including bacterial infections. Shortly after implantation, the surface of Ti implants becomes exposed to bacterial adhesion and biofilm formation. Due to the rich and highly diverse oral microflora, the composition of bacterial biofilm around dental implants is more complex than that on orthopedic implants. *Streptococcus* species play a pivotal role in the early colonization of dental implants, while in the later phases of biofilm formation, the growth of different periodontal pathogens such *as Fusobacterium nucleatum*, *Prevotella intermedia*, *Porphyromonas gingivalis*, and *Aggregatibacter actinomycetemcomitans* has been frequently observed [3,4]. Treatment of biofilm-related infections at implant sites is challenging due to the multispecies composition of microbial biofilm. Moreover, the excessive growth of periodontal pathogens induces a local inflammatory response, often progressing to peri-implantitis and ultimately leading to implant failure [5]. With the increasing prevalence of antibiotic-resistant bacteria, there is interest in antibacterial strategies that do not rely on antibiotics. These include the photoactivation modification of topography and the use of naturally occurring biomolecules like polyphenols [6,7,8].

To address bacterial adhesion on Ti surfaces and reduce inflammation, numerous strategies have been developed, primarily focusing on surface modifications with antimicrobial and/or anti-inflammatory coatings [9].

Whey protein isolate (WPI)-based coatings are promising candidates for the surface modification of Ti implants [10]. One of the main advantages of using WPI in Ti surface modification is its low production cost, as WPI is a by-product of the dairy industry. In addition, WPIs spontaneously form fibrillar structures under acid conditions and high temperature, increasing the surface/volume ratio of the coatings and enhancing cell adhesion and spread [11,12]. Different studies have demonstrated that WPI fibrils have the ability to increase osteogenic differentiation and exhibit antibacterial properties [13,14]. Furthermore, WPI fibrils can be used as matrices for the incorporation of biologically active molecules such as glycosaminoglycans—components of the extracellular matrix (ECM) [15,16]. WPI fibrils have also been successfully modified with biologically active marine-derived polysaccharides, proteins, and polyphenols to enhance their osteogenic properties [17]. Numerous studies have confirmed the osteogenic and anti-inflammatory properties of phloroglucinol (PG), a subunit of marine-derived polyphenols [18,19]. PG has been shown to possess the ability to reduce high levels of oxidative stress, which may be one of the most critical factors contributing to bone resorption [20,21]. The antioxidant, antibacterial, and osteogenic properties of PG were also described by Lišková et al. [22]. More recently, our previous in vitro study confirmed that the incorporation of PG into collagen fibrils promotes osteogenesis and reduces inflammation. PG-enriched collagen fibrillar coatings on Ti surfaces up-regulated the gene expression of osteogenic markers and down-regulated the gene expression of markers involved in pro-inflammatory response in osteoblast-like cells (SaOS-2) [23]. However, despite their numerous advantages, collagen fibrils cannot withstand sterilization by autoclaving, which is a method widely used in the biomedical field.

Two significant advantages of WPI fibrils over collagen fibrils are their much lower cost and ability to withstand sterilization by autoclaving, a clinically accepted and cheap, ubiquitous method. A further ethical advantage is that the use of WPI, being derived from milk, does not involve the slaughter of animals. Therefore, in the following study, WPI fibrils were used as matrices for the incorporation of PG, which is known for its antibacterial, antioxidant, and anti-inflammatory properties, as well as positive impacts on cell adhesion and differentiation [23,24]. To the best of our knowledge, phenolics such as PG have not yet been incorporated into WPI fibril coatings for biomaterial applications. In this context, this work aimed (i) to develop WPI-based fibrillar coatings enriched with PG on Ti6Al4V (Ti) surfaces and (ii) to evaluate the osteogenic and anti-inflammatory properties of these coatings in vitro. The atomic composition of the obtained materials was characterized using X-ray photoelectron spectroscopy (XPS). Human bone marrow-derived mesenchymal stem cells (BM-MSCs) were used to assess the osteogenic differentiation potential of PG-enriched WPI coatings, while the anti-inflammatory activity of the coatings was evaluated in response to a multispecies biofilm challenge. Ti6Al4V samples were produced using electron beam melting (EBM^®^) technology, a 3D printing method where the components are manufactured layer by layer in a vacuum using a metallic precursor powder melted by an intense electron beam, as used in previous work [25]. The use of a 3D printing method to fabricate samples paves the way for the application of this technique with more complicated geometries and internal architectures, which are possible via 3D printing techniques [26].

This study introduces several novel aspects, both biological and material-related. The biological novelty lies in the use of BM-MSCs stimulated with multispecies biofilm, an approach that remains relatively underexplored in the context of biomaterial evaluation. On the material side, this is the first study to use WPI fibrils enriched with PG as a biofunctional coating. Moreover, WPI fibrils are shown to provide a suitable matrix for examining BM-MSC behavior under biofilm challenge. In contrast to our previous work with collagen-based coatings containing PG, this model incorporates biofilm stimulation, enabling a more physiologically relevant assessment in the context of peri-implantitis. WPI coatings also offer great potential for immobilizing bioactive molecules [27], supporting further studies on biofilm–cell–material interactions. 

The current study aims to develop natural, cost-effective, and bioactive Ti coatings for biomaterial applications to improve bone healing and reduce the risk of implant loss due to biofilm-related infections.

## 2. Materials and Methods

### 2.1. Preparation of WPI Fibrillar Suspensions

WPI was provided by BiPro, Davisco Foods International Inc. (BiPro, Davisco Foods International Inc., Eden Prairie, MN, USA). WPI fibril suspensions were prepared as previously described by Keppler et al. [28]. A stock solution of WPI in water was prepared at a concentration of 2.5% *w*/*v* with stirring until complete dissolution. The pH was adjusted to 2.0 by adding a 2 M HCl (Sigma-Aldrich/Merck, Darmstadt, Germany) solution dropwise. A total of 40 mL of WPI solution was heated in a 100 mL Duran glass bottle at 90 °C for 5 h with a stirring speed of 350 rpm to induce fibril formation, resulting in a fibrillar suspension. The WPI fibrillar suspension was then cooled to 4 °C in a water bath to stop the reaction and subsequently stored at 4 °C.

**Figure 1:**
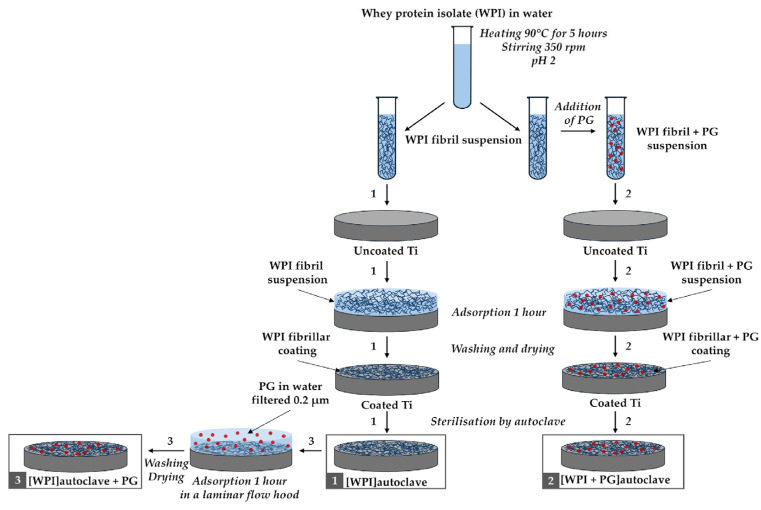
Diagram of the WPI coating process with PG. Three different coating methods are illustrated: (1) WPI coating only, (2) WPI + PG coating autoclaved together, and (3) WPI + PG coating with PG added after sterilization of the WPI coating. Ti: titanium alloy Ti6Al4V; WPI: whey protein isolate; PG: phloroglucinol.

PG (Sigma-Aldrich, Gillingham, UK) was incorporated into the WPI fibrillar suspensions, as illustrated in Figure 1.

After cooling the WPI fibrillar suspension in cold water (4 °C), PG was added to obtain suspensions enriched with PG at two different concentrations: 0.1%, and 0.5% *w*/*v*. Another method for preparing WPI fibril coatings enriched with PG involved the adsorption of PG onto the surface of the WPI fibrillar coatings, as described in Section 2.2 and illustrated in Figure 1.

### 2.2. Preparation and Characterization of WPI Fibrillar Coatings

Ti6Al4V (Ti) discs (10 mm diameter and 2 mm thick) were produced using electron beam melting (EBM^®^) technology, where the components are manufactured layer by layer in a vacuum using a metallic precursor powder melted by an intense electron beam, as previously described [23,29].

The Ti discs were first cleaned with ethanol and water. A WPI fibril suspension was then applied to the Ti6Al4V discs and left for 1 h to allow for fibril adsorption, as shown in Figure 1. After rinsing three times with ultrapure Milli-Q water (Milli-Q, Merck Millipore, Burlington, MA, USA) to remove any non-adhered fibrils and drying at room temperature, the Ti discs were coated with a thin layer of WPI fibrils. Finally, these coatings were sterilized by autoclaving at 121 °C, 1 atm, for 15 min. This procedure is represented as Process 1 in Figure 1. The same procedure was followed to create WPI coatings enriched with PG, as described in Processes 2 and 3 in Figure 1. For Process 2, the WPI fibrillar suspensions containing PG used to coat the Ti discs followed the same steps as described earlier. For Process 3, a PG solution in Milli-Q water (0.2 μm filtered) was added on top of an autoclaved WPI fibrillar coating under sterile conditions. PG adsorption was allowed for 1 h, and the coatings were then washed three times with Milli-Q water and dried at room temperature. Two different concentrations of PG were tested, 0.1% and 0.5% (*w*/*v*) in Milli-Q water. The resulting WPI/PG coatings are named as indicated in Table 1.

The XPS was conducted to analyze the surface chemical composition of all samples, including the uncoated and WPI/PG-coated Ti discs. The analysis was performed using a Axis Supra spectrometer (Kratos Analytical Ltd., Manchester, UK) equipped with a monochromatic Al Kα source (1.487 keV). Briefly, the samples were mounted on a sample holder using carbon tape and an internal flood gun was used for neutralizing the charging effects. Wide-scan spectra were acquired with a pass energy of 160 eV and a step size of 1 eV. Measurements were taken at an emission angle of 0° (relative to the surface normal), with the spectrometer operating at a power of 225 W (15 kV × 15 mA) and analyzing an analysis area of 700 × 300 µm. Three separate locations were analyzed for each sample. The acquired spectra were processed using CasaXPS software (version 2.3.22, Casa Software Ltd., Devon, UK). The binding energies were calibrated against the C-C component of the C1s peak at 284.8 eV to compensate for surface charging effects. The curve-fitting procedure of the components was performed using Gaussian–Lorentzian function with a linear background. The Kratos experimental sensitivity factors were used in the atomic composition calculations.

WPI fibril coatings were visualized on glass substrates by Scanning Electron Microscopy (SEM) (Zeiss EVO MA10; Zeiss, Göttingen, Germany), as described previously [24,30]. The same coating process was used as above. Samples were compared before and after autoclaving.

### 2.3. Bacterial Strains and Culture Conditions

The bacterial strains used in this study were obtained from the Periodontal Research Group culture collection at the Department of Dentistry, University of Birmingham, UK. *Streptococcus mitis* ATCC 49456 and *Aggregatibacter actinomycetemcomitans* ATCC 43718 were cultured for 24 h at 37 °C in a 5% CO_2_ atmosphere on horse blood agar plates (Oxoid, Basingstoke, UK). *Fusobacterium nucleatum* ssp. *polymorphum* ATCC 10593 was grown at 37 °C in an anaerobic chamber (80% N_2_, 10% CO_2_, and 10% H_2_; Don Whitley DG250 Anaerobic Workstation, Don Whitley Scientific, Bingley, UK) on Schaedler Anaerobe agar plates (Sigma-Aldrich/Merck, Darmstadt, Germany) for 48 h. *Porphyromonas gingivalis* ATCC 33277 was cultured anaerobically on horse blood agar plates at 37 °C for at least 96 h.

Liquid cultures of *S. mitis* and *A. actinomycetemcomitans* were grown in Brain Heart Infusion broth (Oxoid, Basingstoke, UK), while *F. nucleatum* and *P. gingivalis* were cultured in Schaedler Anaerobe broth (Oxoid, Basingstoke, UK).

### 2.4. Bacterial Biofilm Development

Multispecies biofilms were prepared as previously described [31]. Briefly, overnight bacterial cultures were standardized to 1 × 10⁷ colony-forming units/mL in artificial saliva (AS), which was prepared as follows: 0.25% *w*/*v* porcine stomach mucins, 0.02% *w*/*v* potassium chloride, 0.02% *w*/*v* calcium chloride dihydrate, 0.2% *w*/*v* yeast extract, 0.5% *w*/*v* proteose peptone (all from Sigma-Aldrich/Merck, Darmstadt, Germany), 0.35% *w*/*v* sodium chloride (Thermo Fisher Scientific, Loughborough, UK), and 0.1% *w*/*v* Lab-Lemco powder (Oxoid, Basingstoke, UK) in Milli-Q water. Urea was added after autoclaving to a final concentration of 0.05% *v*/*v* (Sigma-Aldrich, Gillingham, UK).

To initiate biofilm formation, the standardized *S. mitis* culture was added to a 24-well plate containing 13 mm Thermanox™ coverslips (Thermo Fisher Scientific, Loughborough, UK) and incubated at 37 °C with 5% CO_2_. After 24 h, the biofilm supernatant was removed, and the standardized *F. nucleatum* culture was added. The biofilms were then further incubated under anaerobic conditions at 37 °C for another 24 h. The supernatant was removed, and *A. actinomycetemcomitans* and *P. gingivalis* were added. The biofilms were incubated anaerobically for an additional 4 days at 37 °C, with AS replaced every 24 h.

### 2.5. Analysis of Bacterial Biofilm Structure and Composition

The structure of bacterial biofilm was analyzed using scanning electron microscopy (SEM). For SEM imaging, biofilm samples were prepared as previously described [32]. Initially, the biofilms were fixed using 2.5% glutaraldehyde (Agar Scientific, Stansted, UK) in a 0.1 M sodium cacodylate buffer (pH 7.4, BioWorld, Dublin, Ireland) for 10 min at room temperature. Subsequently, the biofilms were dehydrated through increasing concentrations of ethanol (20–100%) (Sigma-Aldrich, Gillingham, UK). Next, the biofilms were treated with hexamethyldisilazane (HMDS; Sigma-Aldrich/Merck, Darmstadt, Germany) as a drying agent. After overnight evaporation of the HMDS, the biofilm samples were mounted onto aluminum specimen stubs (Agar Scientific, Stansted, UK), sputter-coated with gold, and visualized using a scanning electron microscope (Zeiss EVO MA10; Zeiss, Göttingen, Germany).

The composition of bacterial biofilm was analyzed using real-time quantitative PCR (qPCR). Briefly, the bacterial biofilm was scraped from the coverslip surface using standard cell scrapers (Thermo Fisher Scientific, Loughborough, UK) and resuspended in 180 μL of digestion buffer (Invitrogen, Carlsbad, CA, USA). Total bacterial DNA was then isolated using the PureLink Genomic DNA kit (Invitrogen, Carlsbad, CA, USA) following the manufacturer’s instructions. The quality and quantity of the extracted DNA were assessed using a NanoDrop spectrophotometer (Thermo Fisher Scientific, Loughborough, UK). Extracted DNA was amplified using primers targeting the 16S ribosomal RNA (rRNA) gene specific to each bacterial species. The primers used were the same as those described previously [33]. The primer sequences are presented in Table 2.

Briefly, 2.5 μL of total DNA (100 ng) was added to a reaction mix containing 7.5 μL of GoTaq qPCR Master Mix (Promega, Madison, WI, USA), 0.5 μL (10 μM) of each primer (GenoMed, Warsaw, Poland), and 4 μL of H_2_O (Promega, Madison, WI, USA). PCR amplification was conducted under the following conditions: 95 °C for 10 min, followed by 40 cycles of 95 °C for 15 s, 56 °C for 45 s, and 72 °C for 30 s, with a final extension at 72 °C for 5 min. The number of each bacterium was determined using a standard curve prepared from serial dilutions of DNA isolated from fresh cultures of each bacterial species.

### 2.6. BM-MSC Culture and Seeding

This study was performed using commercially available human bone marrow-derived mesenchymal stem cells (BM-MSCs; catalog no. PCS-500-012) obtained from the American Type Culture Collection (ATCC; Manassas, VA, USA). The cells were cultured in an α-minimum essential medium (α-MEM) (Lonza, Verviers, Belgium) supplemented with 10% fetal bovine serum (FBS) (Invitrogen, Paisley, UK), 100 U/mL penicillin (Sigma-Aldrich, Gillingham, UK), and 100 μg/mL streptomycin (Sigma-Aldrich, Gillingham, UK) and incubated at 37 °C in a humidified atmosphere of 95% air and 5% CO_2_ until confluence was reached. BM-MSCs at passages 3–5 were used in the present study. For all in vitro assays, BM-MSCs were seeded directly onto uncoated Ti discs, Ti discs coated with WPI alone ([WPI] autoclave), or Ti discs coated with WPI enriched with PG at various concentrations ([WPI + PG 0.1%] autoclave, [WPI + PG 0.5%] autoclave, [WPI] autoclave + PG 0.1%, [WPI] autoclave + PG 0.5%), as described earlier in Section 2.2 and summarized in Table 1. The discs were placed in wells of 48-well tissue culture polystyrene (TCPS) plates (Life technologies, Paisley, UK) at a density of 3 × 10^4^ cells per disc and cultured for 72 h.

### 2.7. BM-MSCs: Bacterial Biofilm Challenge

After 72 h of BM-MSC cultivation in a standard medium, each Ti disc with BM-MSCs was carefully removed from the wells of a 48-well TCPS plate using sterile forceps and transferred to the wells of a 24-well TCPS plate (Life technologies, Paisley, UK). The BM-MSCs on the Ti discs were washed twice with a fresh medium and then subsequently incubated for 2 h in an antibiotic-free medium at 37 °C in a 5% CO_2_ atmosphere, with one biofilm-coated glass coverslip per well. The glass coverslips were positioned on the ring supports, with the biofilm-facing side oriented toward the titanium surface, according to the co-culture set-up, as previously described [34]. The distance between the BM-MSC layer and the biofilm-coated coverslip was maintained to allow for fluid flow. After 2 h, the biofilm-coated glass coverslips were removed from all wells, and the Ti discs with BM-MSCs were washed twice with a fresh medium. The Ti discs were then cultured for 48 h in a standard medium with antibiotics for further in vitro assays, including metabolic activity measurement, morphology assessment, and gene expression analysis.

For this study, two experimental groups were established: (i) the unstimulated control group, consisting of BM-MSCs cultured on Ti with and without WPI/PG coatings under standard conditions, without exposure to the biofilm, and (ii) the biofilm-stimulated group, in which BM-MSCs were exposed to biofilm for 2 h under co-culture conditions, as described above.

### 2.8. SEM Evaluation of BM-MSCs on Ti6Al4V Alloy

SEM analyzes were conducted to examine the attachment, spread, and morphology of BM-MSCS cultured on the surface of tested Ti discs. Briefly, after 72 h of cell cultivation, the samples were washed three times with phosphate-buffered saline (PBS) for 10 min to remove non-adherent cells. The cells were then fixed with 2% glutaraldehyde (Agar Scientific, Stansted, UK) in 0.1 M sodium cacodylate buffer (pH 7.4) (Sigma-Aldrich, Gillingham, UK) for 2 h at room temperature. Following the removal of the glutaraldehyde solution, the samples were sequentially dehydrated in increasing concentrations of ethanol. Finally, hexamethyldisilane (HMDS; Sigma-Aldrich, Gillingham, UK) was added to each sample and allowed to dry overnight at room temperature. Imaging was performed using a scanning electron microscope (Zeiss, Göttingen, Germany).

### 2.9. BM-MSC Metabolic Activity

The metabolic activity of BM-MSCs was determined 48 h after biofilm stimulation. Metabolic activity was assessed in both unstimulated and biofilm-stimulated BM-MSCs in parallel. Briefly, the cell culture medium was replaced with fresh medium containing methylthiazolyldiphenyl-tetrazolium bromide (MTT) (Sigma-Aldrich, Gillingham, UK) at a final concentration of 0.5 mg/mL. After 3 h of incubation at 37 °C in a humidified CO_2_ incubator, the medium containing MTT was removed and isopropanol with 0.04 N HCl (Sigma-Aldrich, Gillingham, UK) was added to dissolve the formazan crystals. The absorbance was measured at 570 nm. All experiments were performed four times in duplicate (n = 8).

### 2.10. BM-MSC Gene Expression Analysis

To determine gene expression, total RNA was isolated after 48 h from both unstimulated and biofilm-stimulated BM-MSCs using TRI reagent (Sigma-Aldrich, Gillingham, UK) and the RNeasy Mini Kit (Qiagen, Crawley, UK), following the manufacturer’s protocol. The purity and quantity of RNA were measured using a NanoDrop spectrophotometer (Thermo Fisher Scientific, Loughborough, UK). The RNA was reverse-transcribed to cDNA using a one-step high-capacity cDNA Reverse Transcription (RT) kit (Applied Biosystems, Warrington, UK) according to the manufacturer’s instructions. The qPCR was conducted on a CFX96 Touch Real-Time PCR Detection System (BioRad, Feldkirchen, Germany) using Roche SYBR Green PCR Master Mix (Roche Diagnostics GmbH, Mannheim, Germany). PCR reactions were performed in 10 µL volumes in a 96-well plate (Roche Diagnostics GmbH, Mannheim, Germany), with each reaction containing 1 µL of cDNA and 9 µL of the reaction mixture, as per the manufacturer’s specifications. All samples were amplified in duplicates. The PCR conditions included an initial denaturation step at 95 °C for 5 min, followed by 40 cycles of 95 °C for 10 s, 60 °C for 15 s, and 72 °C for 20 s. The primer sequences (Sigma-Aldrich, Gillingham, UK) for specific target genes are listed in Table 3. Glyceraldehyde-3-phosphate dehydrogenase (*GAPDH*) was used as the reference gene in each experiment. The relative quantification of mRNA levels of the target genes was analyzed using the comparative CT (threshold cycle) method (2^−ΔΔCt^), as previously described by Livak et al. Relative expression levels were calculated for each sample after normalization against the reference gene. All experiments were performed four times in duplicate (n = 8).

### 2.11. Statistical Analysis

The data are presented as mean values ± standard deviation (SD) or standard error of the mean (SEM). Statistical differences in the in vitro studies were calculated using one-way ANOVA, followed by a multiple comparison Bonferroni test, performed with SPSS version 22 (IBM, Armonk, NY, USA). A *p* value < 0.05 was considered statistically significant.

## 3. Results and Discussion

### 3.1. Characterization of the WPI/PG Coatings on Ti6Al4V Alloy

**Figure 2:**
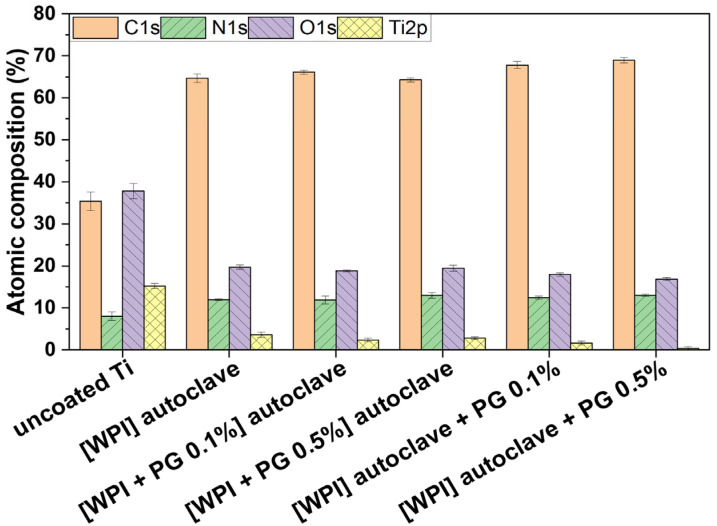
Atomic composition of uncoated Ti and WPI coatings without PG, with 0.1% of PG and 0.5% of PG. [WPI + PG 0.1%] autoclave and [WPI + PG 0.5%] autoclave mean that the coatings containing PG were autoclaved. [WPI] autoclave + PG 0.1% and [WPI] autoclave + PG 0.5% mean that only the WPI coating was autoclaved; PG was added afterwards using a sterile filter and syringe. Error bars represent SD. Ti: titanium alloy Ti6Al4V; WPI: whey protein isolate; PG: phloroglucinol.

The presence of WPI/PG coatings on Ti was confirmed using XPS analyses. As shown in Figure 2, all coatings were mainly constituted of carbon (C), nitrogen (N), and oxygen (O), which demonstrates the presence of the protein layer. Indeed, nitrogen and carbon were detected from the protein containing primary amine (-NH2) and carboxyl (-COOH) functional groups. Moreover, the decrease in %Ti from 15% (uncoated Ti) to less than 4% confirmed the presence of the coating. As Ti signal was still detected in the presence of WPI fibrils, the coating may be either thinner than 15 nm, which is the depth detection limit of XPS, or non-uniform revealing gaps between the fibrils. No significant differences were observed among the different coatings. However, the Ti content decreased significantly from 3.6% (WPI) to 0.4% ([WPI] autoclave + PG 0.5%) which indicates that the coating may be thinner or more uniform. PG was not easily detectable, as oxygen and carbon signals may come from both the protein and Ti substrate.

SEM images of the coatings (Figure S1, Supplementary Information) demonstrated the presence of fibrils and that fibrils withstood autoclaving, as demonstrated in a previous work [21]. The presence of PG did not appear to influence the fibril thickness, which was approximately 20 microns on all samples. The fibril layers appeared to be approximately 1 fibril thick and covered most of the sample surfaces, which is in agreement with the XPS results (Figure 2). As WPI fibrils are large molecules which withstand autoclaving and have a very high surface area/volume ratio, it is expected that the coatings will be stable under physiological conditions, in the same way that collagen fibril coatings are known to be stable. The fibril thicknesses observed in this study are consistent with those observed in previous works [24,27]. The fact that PG did not influence fibril thickness suggests that it is not incorporated into fibrils during fibril formation and binds to the surface of already formed fibrils. Alternatively, if it is incorporated into fibrils, being a small molecule, its presence may not impede fibril formation, or does so not significantly. This finding is similar to that observed in our previous work on collagen fibril coatings containing PG [23], where no marked change in morphology of collagen fibrils was observed due to the presence of PG.

### 3.2. Characterization of the Multispecies Biofilm

**Figure 3:**
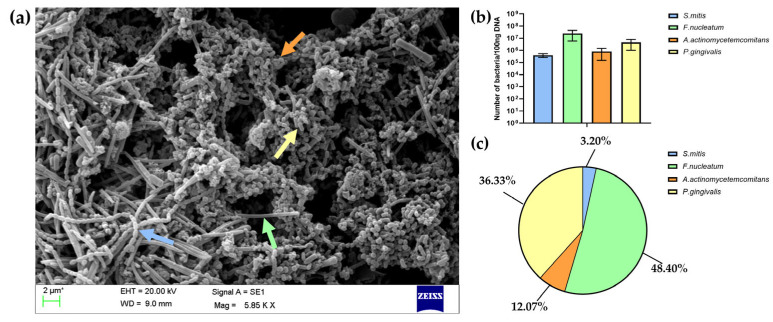
(**a**) Representative scanning electron micrograph of a multispecies biofilm formed on a glass coverslip, subsequently used in the co-culture model with BM-MSCs. Arrows in the SEM image indicate examples of four different bacterial species: *S. mitis* (blue arrow), *F. nucleatum* (green arrow), *P. gingivalis* (yellow arrow), and *A. actinomycetemcomitans* (orange arrow). (**b**) The number of each bacterial species quantified using the qPCR method and calculated per 100 ng of DNA isolated from the biofilm. (**c**) The results of bacterial quantification presented as the percentage (%) of the total number of bacteria in the biofilm.

The architecture of biofilm after 5 days of cultivation was assessed using scanning electron microscopy (SEM). As shown in Figure 3a, the biofilm showed the formation of a 3D structure with spatial heterogeneity in its bacterial arrangement. Co-aggregates of *P. gingivalis*, *A. actinomycetemcomitans*, *F. nucleatum*, and *S. mitis* were observed across the entire surface. These species were identified in the SEM micrograph based on their distinct morphological features: *S. mitis* appeared as spherical cells arranged in long chains; *F. nucleatum* as elongated and slender rods; *P. gingivalis* as short rods; and *A. actinomycetemcomitans* as small, oval-shaped coccobacilli [35,36,37,38].

Additionally, a SEM analysis confirmed the presence of extracellular polymeric substances (EPSs), which form a sticky matrix embedding the bacteria and contributing to biofilm stabilization. The quantification of each bacterial species in the mature biofilm was performed using the qPCR method. The results presented in Figure 3b,c indicated the dominance of *F. nucleatum* within the mature biofilm, followed by *P. gingivalis*, with smaller contributions from *A. actinomycetemcomitans* and *S. mitis*. The obtained proportions of bacteria reflect the composition of pathogenic oral biofilm associated with peri-implant infections, where increases in the number of pathogenic bacteria, such as *F. nucleatum* and *P. gingivalis*, are observed along with a reduction in the number of *Streptococcus* species [3,4,5].

The established multispecies biofilm was subsequently used in an in vitro co-culture model with human BM-MSCs. In contrast to many previous studies focusing on single bacterial species or isolated virulence factors such as lipopolysaccharide (LPS) to mimic peri-implant infections, the present study employed a complex, multispecies biofilm that closely reflects the polymicrobial nature of peri-implantitis [31,39,40]. Using this model, WPI/PG coatings were evaluated for their potential to modulate the BM-MSC response under biofilm challenge. By incorporating these coatings into the co-culture model, this study aimed to assess not only their osteogenic properties but also their ability to support BM-MSC function in the presence of a biofilm microenvironment.

### 3.3. In Vitro Studies: Evaluation of WPI/PG Coatings in a Co-Culture Model of Multispecies Biofilm and BM-MSCs

In the present study, the osteogenic and anti-inflammatory properties of WPI/PG coatings on Ti were evaluated using BM-MSCs. These cells can undergo differentiation into multiple cell types and modulate host-immune responses by the secretion of pro-healing factors, including growth factors, cytokines, and chemokines [41,42]. However, due to the presence of oral pathogenic biofilms, the integration of Ti implant with bone tissue can be affected [3]. Therefore, in this work, BM-MSCs were stimulated with multispecies biofilm to investigate the effect of the coatings on unstimulated and biofilm-stimulated cell behavior.

#### 3.3.1. BM-MSC Attachment, Spread, and Morphology

**Figure 4:**
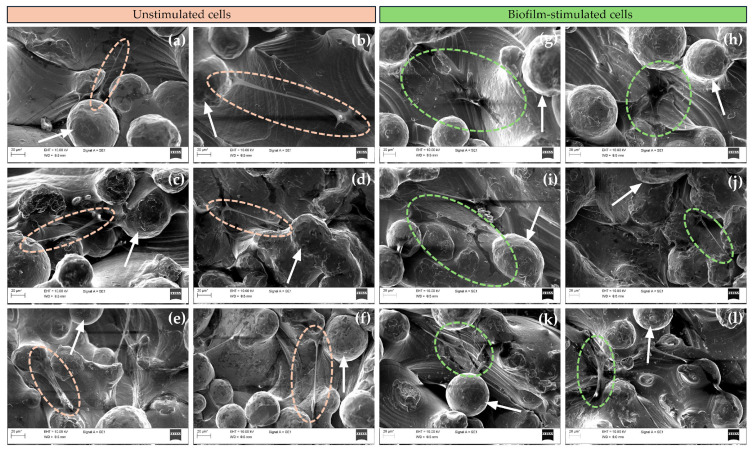
Representative SEM images of unstimulated and biofilm-stimulated BM-MSCs on (**a**,**g**) uncoated Ti6Al4V; (**b**,**h**) WPI; (**c**,**i**) [WPI + PG 0.1%] autoclave; (**d**,**j**) [WPI + PG 0.5%] autoclave; (**e**,**k**) [WPI] autoclave + PG 0.1%; and (**f**,**l**) [WPI] autoclave + PG 0.5% coatings on Ti6Al4V. Ellipses indicate unstimulated cells (orange) and biofilm-stimulated cells (green) and white arrows show particles of Ti6Al4V powder. Scale bar: 20 μm. Ti: titanium alloy Ti6Al4V; WPI: whey protein isolate; PG: phloroglucinol.

The implant surface determines the morphology of MSCs, which is a key indicator of cell adhesion and differentiation [43]. SEM images were obtained after 72 h of BM-MSC culture to assess the differences in cell attachment, spread, and morphology on uncoated Ti and WPI-coated Ti, with and without PG (Figure 4).

For all tested surfaces, BM-MSCs were located in the pores between the particles of Ti powder. Moreover, the cells appeared to spread well on all coating types, showing elongated morphology and displaying numerous filopodia. No significant differences in cell morphology or spread between the sample groups were observed. These results indicate that cell attachment, spread, and morphology depend more on the topography of Ti discs than on the incorporated WPI coatings. Numerous studies have shown the positive effect of porous surfaces on cell adhesion [44,45], and our results clearly demonstrate that the cells adhered well to all porous Ti surfaces and developed numerous filopodia, indicating good affinity with the Ti, regardless of WPI coatings [46]. The morphology of BM-MSCs was also assessed in response to biofilm stimulation. After biofilm exposure, BM-MSCs cultured on uncoated Ti and WPI coatings without PG showed a more flattened morphology, whereas on WPI coatings enriched with different concentrations of PG, most cells demonstrated an elongated spindle shape, similar to the morphology observed in unstimulated BM-MSCs. These results suggest a possible protective role of PG incorporated into WPI coatings for BM-MSCs upon biofilm exposure. This observation is particularly relevant in the context of peri-implantitis, where local inflammation and bacterial colonization often compromise the regenerative potential of MSCs. The ability of PG-enriched WPI coatings to maintain the typical spindle-like morphology of BM-MSCs suggests a modulatory effect of these coatings on their response to biofilm-associated inflammation, potentially by limiting cytoskeletal rearrangements. Additionally, the flattened morphology observed under biofilm challenge in cells on uncoated Ti surfaces or those coated with PG-free WPI may reflect a stress-induced phenotype or an early sign of impaired function of BM-MSCs. It has been reported that a large, flat morphology is more typical of the inflammatory phenotype of BM-MSCs, while the spindle-shaped form corresponds to osteoblastic morphology [47]. The protective effect of PG may result from its antioxidant, anti-inflammatory, or antimicrobial properties [22].

Together, these results indicate that PG-enriched WPI coatings actively protect BM-MSC morphology under inflammatory conditions, helping maintain their regenerative capacity. This supports their potential as bioactive surface coatings to improve implant outcomes in peri-implantitis.

#### 3.3.2. BM-MSC Metabolic Activity

**Figure 5:**
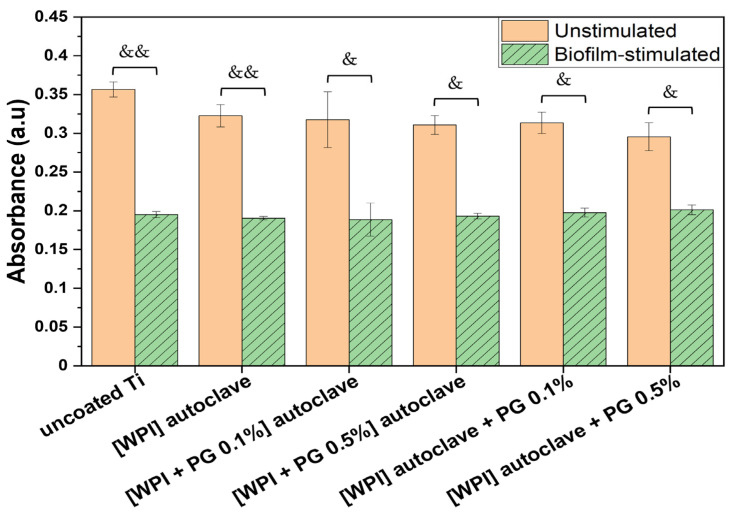
Metabolic activity of unstimulated and biofilm-stimulated BM-MSCs after 48 h using MTT test. BM-MSCs were stimulated with biofilm for 2 h, and metabolic activity was analyzed directly after biofilm stimulation. The results are shown as mean (n = 4, two technical repetitions), and bars represent SEM. Significant differences for unstimulated vs. biofilm-stimulated BM-MSCs are indicated with & (*p* < 0.05) and && (*p* < 0.01). Ti: titanium alloy Ti6Al4V; WPI: whey protein isolate; PG: phloroglucinol.

The metabolic activity of unstimulated and biofilm-stimulated BM-MSCs was assessed after 48 h, as summarized in Figure 5.

The results showed a significant decrease in metabolic activity when cells had been stimulated with biofilm. This may be due to the release of soluble factors from the biofilm, which impact the cell function, as already mentioned in a previous study [48]. They showed that mesenchymal stromal cells exposed to *Staphylococcus aureus* and *Pseudomonas aeruginosa* biofilm media exhibited reduced cell viability due to apoptosis activation, as well as reduced migration and differentiation abilities. Other studies have investigated this effect, particularly in the context of chronic wounds, where biofilm is considered to contribute to wound chronicity [49].

Although biofilm stimulation led to reduced metabolic activity across all tested groups, the extent of the reduction varied depending on the surface. In the absence of biofilm, all surfaces—including uncoated Ti, WPI, and WPI enriched with either 0.1% or 0.5% PG—supported similarly high levels of metabolic activity, indicating that neither the WPI coating nor the addition of PG compromised BM-MSC viability. Upon biofilm exposure, the most pronounced reduction was observed on uncoated Ti (approximately 50% decrease), followed by WPI alone, which also showed a marked decrease. Interestingly, the WPI coatings supplemented with PG resulted in an attenuated decrease, indicating a protective effect. Notably, in the presence of 0.5% PG, biofilm-stimulated BM-MSCs exhibited a smaller decrease in metabolic activity compared to those cultured on 0.1% PG, indicating a dose-dependent effect. These findings suggest that PG not only maintains BM-MSC viability under normal conditions but may also mitigate biofilm-induced metabolic impairment.

#### 3.3.3. Expression of Genes Related to Bone Matrix Formation and Mineralization in BM-MSCs

**Figure 6:**
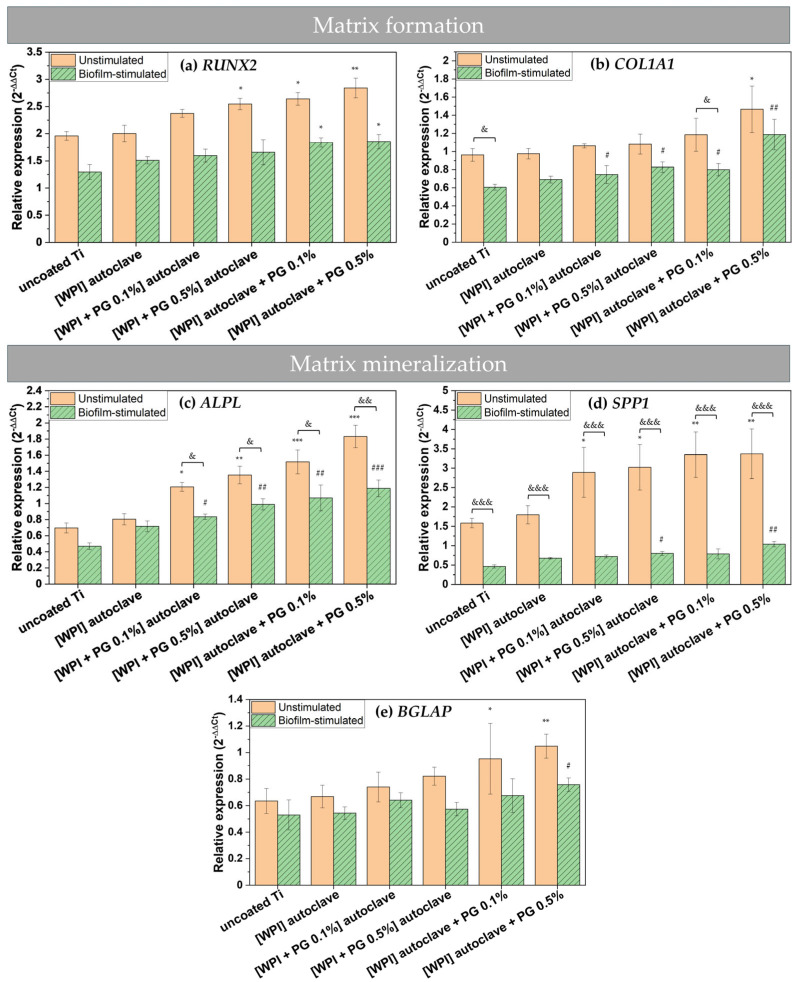
Relative gene expression for matrix formation markers—(**a**) *RUNX2*—and (**b**) *COL1A1* and matrix mineralization markers—(**c**) *ALPL*, (**d**) *SPP1*, and (**e**) *BGLAP*. The results are shown as means (n = 4, two technical repetitions), and bars represent SEM. * and # represent statistical analyses between uncoated Ti and tested samples for unstimulated cells and biofilm-stimulated cells, respectively. (*, #, & *p* < 0.05; **, ##, && *p* < 0.01; ***, ###, &&& *p* < 0.001). Ti: titanium alloy Ti6Al4V; WPI: whey protein isolate; PG: phloroglucinol.

The gene expression of bone matrix formation markers was analyzed in unstimulated and biofilm-stimulated cells. Runt-related transcription factor 2 (*RUNX2*) is an early marker of bone matrix formation, as it is expressed in preosteoblast cells, while collagen type I alpha 1 chain (*COL1A1*) is known as a key marker of bone matrix production [50,51]. Both genes are important markers of the early stages of bone–implant integration. As illustrated in Figure 6a, the incorporation of PG into WPI coatings resulted in an up-regulation of *RUNX2* expression in unstimulated BM-MSCs. This finding is highly significant, as RUNX2 is considered the most important transcription factor in osteogenesis, playing a pivotal role in the early differentiation of BM-MSCs. Numerous studies have demonstrated that the overexpression of *RUNX2* induces the differentiation of BM-MSCs into osteoblasts and activates osteogenesis-related genes such as collagen type I, osteopontin, and bone gamma-carboxyglutamate protein [52,53]. Our results indicate that WPI/PG coatings promote the osteogenic differentiation of BM-MSCs by up-regulating *RUNX2* expression. However, an elevated expression of *RUNX2* can inhibit cell proliferation by promoting cell cycle arrest in the G0/G1 phase [52]. MTT results (Figure 5), which reflect both metabolic activity and cell proliferation, showed that WPI/PG coatings slightly reduced BM-MSC proliferation, but the differences were not statistically significant. This suggests that PG incorporation into WPI coatings, at the tested concentrations, can significantly enhance BM-MSC differentiation without impairing proliferation.

An RT-qPCR analysis of *COL1A1* expression further confirmed the pro-osteogenic potential of PG-enriched WPI coatings. A significant up-regulation of *COL1A1* was observed in BM-MSCs cultured on these coatings, with expression levels positively correlating with the concentration of PG (Figure 6b). These results are in accordance with our previous study on PG incorporated into collagen fibrillar coatings, where *COL1A1* expression was also studied [23]. Notably, the highest *COL1A1* expression was detected in cells grown on coatings containing the highest amount of PG without being autoclaved. This indicates that PG was more biologically active without being sterilized by autoclaving and the heat may impact its activity.

Other studies have also indicated that the addition of PG into WPI hydrogels supports the growth and collagen production of human dental pulp stem cells (DPSCs), promoting the formation of the ECM [19]. Given that COL1A1 is an abundant protein in the native ECM and plays a crucial role in maintaining the structural integrity of newly formed bone, our findings highlight the potential of WPI/PG coatings to support early matrix deposition. Moreover, the observed increase in *COL1A1* expression may be directly linked to higher *RUNX2* levels, as RUNX2 is known to regulate the transcription of *COL1A1* [53]. Together, these results point to a synergistic effect of WPI/PG coatings in enhancing osteogenic differentiation and matrix formation, which is essential for successful bone–implant integration.

As shown in Figure 6a, biofilm stimulation significantly down-regulated the expression of *RUNX2* on all examined Ti surfaces. The expression of *COL1A1* also appeared to be reduced in biofilm-stimulated cells compared to unstimulated cells, but the differences were not statistically significant (Figure 6b). Notably, in the presence of PG-enriched WPI coatings, the expression of these genes in biofilm-stimulated cells increased compared to uncoated Ti, particularly when PG was not autoclaved and was present at a concentration of 0.5%. These findings suggest that PG-enriched WPI coatings may help maintain osteogenic potential even under peri-implantitis-like conditions.

The persistent colonization of implant surfaces by pathogenic bacteria can strongly inhibit the osteogenic differentiation of BM-MSCs [39]. However, based on *RUNX2* and *COL1A1* expression profiles in infected BM-MSCs, WPI/PG coatings could sustainably promote osteogenic differentiation even during infection. Future studies should examine whether WPI/PG coatings reduce matrix metalloproteinase (MMP) expression in infected BM-MSCs, since elevated MMPs, particularly those degrading collagen I and III, impair bone matrix formation [54]. Thus, reducing the MMP level could prevent matrix breakdown and support regeneration upon peri-implantitis.

Here, we investigated also the effect of PG-enriched WPI coatings on the mineralization potential of BM-MSCs by measuring the gene expression of osteogenic markers. The results showed that WPI coatings with PG promoted the mRNA expression of alkaline phosphatase (*ALPL*), osteopontin (*SPP1*), and bone gamma-carboxyglutamate protein (*BGLAP*) in both unstimulated and biofilm-stimulated cells. These findings are important, as *ALPL* is an essential marker for the bone mineralization of osteoblastic cells [55], *SPP1* is a key marker of osteogenic differentiation [56], and *BGLAP* is involved in the regulation of the mineralization process [57].

The expression of all osteogenic genes increased in the presence of PG. Similar results were observed in our previous study on PG using human osteosarcoma cell line SaOS-2, where the gene expression of osteogenic markers was evaluated [23]. Moreover, our findings demonstrate a clear, dose-dependent pro-osteogenic activity of PG. Without biofilm stimulation, 0.5% PG caused the most pronounced induction of *ALPL* and *SPP1* (Figure 6c,d), whereas the effect on the late marker *BGLAP* was more limited, but still significant. This attenuated response is likely due to the early measurement time point (day 3), as *BGLAP* typically peaks between days 14 and 21 in osteoblast-lineage cultures, so the capacity of WPI/PG coatings to up-regulate this late marker may be under-represented here [58,59]. Incorporating PG after autoclaving further enhanced osteogenic responses, implying that heat reduces but does not eliminate PG activity.

Biofilm stimulation markedly reduced osteogenic gene expression—particularly *SPP1*—on all Ti surfaces. Nevertheless, PG-enriched WPI coatings, especially the non-autoclaved 0.5% PG variant, maintained elevated *ALPL* expression under biofilm stimulation, indicating that PG/WPI coatings form a protective interface that preserves osteogenic activity despite microbial challenge. The up-regulation of osteogenic markers in both unstimulated and biofilm-stimulated BM-MSCs aligns with increased *RUNX2* levels: PG increases *RUNX2*, which then activates its downstream transcriptional targets, first driving matrix deposition (*COL1A1*) and later mineralization (*ALPL*, *SPP1*, and *BGLAP*).

Overall, the results suggest that PG-enriched WPI coatings on titanium may promote bone formation and mineralization by enhancing the expression of osteogenic markers, even in the presence of implant-related biofilm infection. The enhanced osteogenesis observed in response to PG-enriched WPI coatings could potentially improve bone–implant integration and reduce the risk of implant failure. However, WPI/PG coatings may alter surface properties, such as roughness, which may also contribute to the increased expression of osteogenic markers. Therefore, future studies should provide a detailed physicochemical characterization of these properties and correlate them with in vitro results to clarify how each parameter contributes to the observed pro-osteogenic effect.

#### 3.3.4. Gene Expression of Pro-Inflammatory Markers in BM-MSCs

**Figure 7:**
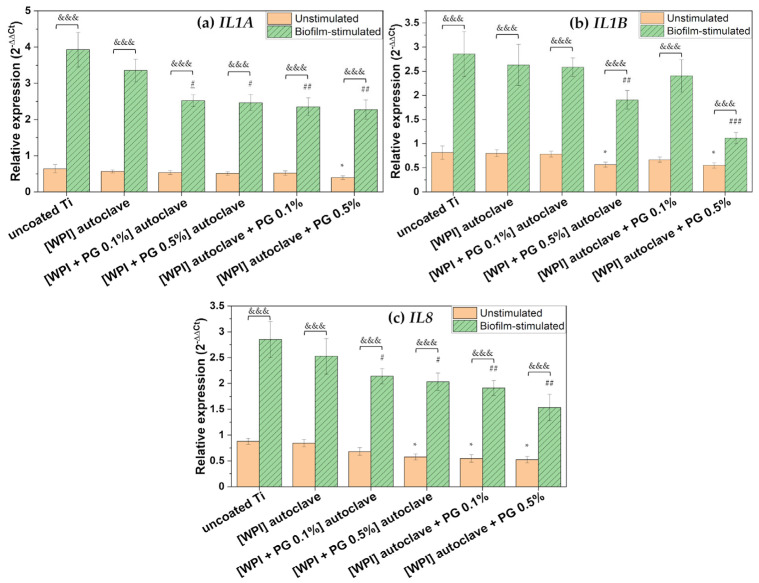
Relative gene expression of pro-inflammatory markers: (**a**) *IL1A*, (**b**) *IL1B*, and (**c**) *IL8*. The results are shown as means (n = 4, two technical repetitions), and bars represent SEM. * and # represent significant differences between uncoated Ti and tested samples for unstimulated cells and biofilm-stimulated cells, respectively. (*, # *p* < 0.05; ## *p* < 0.01; ###, &&& *p* < 0.001). Ti: titanium alloy Ti6Al4V; WPI: whey protein isolate; PG: phloroglucinol.

The implantation of biomaterials, such as Ti implants, into bone may trigger host responses, including excessive inflammation, which can interfere with the bone-healing process. Notably, the secretion of inflammatory cytokines is not restricted to immune cells alone. MSCs recruited to the implantation site are also capable of producing a broad range of cytokines, including interleukins, which are involved in the regulation of inflammation and osteogenesis [60]. In this study, the gene expression of selected pro-inflammatory markers, interleukin-1α (*IL1A*), interleukin-1β (*IL1B*), and interleukin-8 (*IL8*), was evaluated, as shown in Figure 7.

WPI-containing PG exhibited a general significant down-regulation of pro-inflammatory markers in unstimulated BM-MSCs compared to uncoated Ti. Moreover, the results indicated that WPI coatings containing a high amount of PG (0.5%) added after autoclaving down-regulated the gene expression of *IL1B* and *IL8*, suggesting that PG-enriched WPI coatings may contribute to the attenuation of the inflammatory response.

IL-8 plays a pivotal role in the recruitment of neutrophils to the site of implantation [61]. Although neutrophils are essential during the early stages of healing, their prolonged activation, especially under continuous stimulation by microbial factors, can lead to the release of MMPs and other degradative enzymes that contribute to bone damage and may compromise implant integration [54]. The observed down-regulation of *IL8* in BM-MSCs cultured on WPI/PG-coated surfaces may therefore reflect a reduction in neutrophil chemoattractant signaling, potentially mitigating excessive inflammation. IL-1β is also a key mediator of inflammation that has been associated with bone resorption and impaired osteogenic differentiation of MSCs. High and persistent expression of *IL1B* at the implant site inhibits osteogenesis, stimulates osteoclastogenesis, and disrupts implant integration. Therefore, the reduced expression of *IL1B* in response to WPI/PG coatings is significant, as it may contribute to a more favorable inflammatory environment for bone–implant integration. Importantly, this suppressive effect on *IL1B* was observed only in the presence of PG, as WPI coatings alone did not induce any changes. Furthermore, the inhibitory effect was evident at the higher PG concentration of 0.5%, while the lower concentration of 0.1% did not affect *IL1B* expression. As already described in the literature, PG may prevent inflammation by down-regulating the gene expression of inflammatory markers, such as TNF-α, IL-1β, IL-6, and prostaglandin E_2_, through a potential reaction mechanism between hydroxyl groups and reactive oxygen species [21]. These findings are in line with our previous study, where a similar anti-inflammatory effect of PG was observed on collagen fibrillar coatings [23].

No significant change in *IL1A* expression was observed in unstimulated BM-MSCs, suggesting that PG’s immunomodulatory action could be selective.

As shown in Figure 7, BM-MSCs showed high expression of pro-inflammatory markers in response to oral bacterial biofilm challenge. Numerous studies have reported that complex multispecies biofilms stimulate various types of cells involved in bone–implant integration to express and release a variety of inflammatory mediators, including IL-1β, IL-6, and IL-8 [34,62,63]. The higher expression of these inflammatory mediators during biofilm-related peri-implant infections contributes to both soft and hard tissue damage [3,64]. In the present study, the bacterial biofilm used to challenge BM-MSCs was dominated by *F. nucleatum* and *P. gingivalis*, as shown in Figure 3. These bacteria are known to produce a range of virulence factors, including proteolytic enzymes, which are released into the culture medium and may strongly stimulate a pro-inflammatory response in BM-MSCs [31]. However, our results indicated that WPI coatings enriched with PG significantly reduced the expression of key pro-inflammatory markers in BM-MSCs challenged with biofilm. A marked down-regulation of *IL1A* was observed on PG/WPI-coated surfaces—regardless of PG concentration—compared to uncoated Ti. Interestingly, this effect was not observed in unstimulated BM-MSCs, likely due to the low basal *IL1A* expression, which possibly makes PG’s effect undetectable without pro-inflammatory stimulation.

Moreover, PG incorporation into WPI coatings resulted in a dose-dependent decrease in the expression of *IL1B* and *IL8*, with the 0.5% PG concentration showing a more pronounced inhibitory effect than the 0.1% concentration. This finding is particularly relevant, as IL-1β is a well-recognized marker of peri-implantitis and is frequently detected at high levels in peri-implant crevicular fluid. Similarly, IL-8 is known to contribute to the pro-inflammatory environment at the implant surface, promoting osteoclast differentiation, eventually accelerating bone resorption and compromising implant stability [65,66].

Importantly, our results also demonstrated that autoclaving PG could compromises its immunomodulatory properties. In the case of *IL1B* and *IL8*, coatings containing heat-sterilized PG exhibited reduced ability to suppress pro-inflammatory marker expression, indicating a possible thermal degradation of PG functional groups, which may impair its bioactivity. Therefore, non-thermal sterilization methods should be considered to maintain its biological function.

Taken together, these findings suggest that PG-enriched WPI coatings may help attenuate biofilm-induced inflammation by down-regulating key mediators such as *IL1A*, *IL1B*, and *IL8*. This anti-inflammatory effect appears to be dose-dependent and to occur primarily under inflammatory conditions.

Furthermore, given that phenolic compounds like PG exhibit broad-spectrum antimicrobial activity [67,68,69], it is possible that the reduced inflammatory response observed here may also be partially attributed to antimicrobial effects. Future studies should evaluate the antibacterial properties of WPI/PG coatings and include protein-level analyses, such as quantitative proteomics, to better explain the mechanisms underlying their anti-inflammatory activity.

### 3.4. General Discussion and Outlook

Further work should focus on adhesion studies and possible protein release from coatings, as well as the possible effects of autoclaving on adhesion or protein release. Another future research topic is a comparison of PG with other bioactive molecules of plant origin, such as flavonoids, tannins, or other polyphenols.

## 4. Conclusions

This study demonstrated that the surface modification of Ti6Al4V alloy with WPI/PG coatings effectively enhances the interaction between BM-MSCs and titanium, even in the presence of pathogenic multispecies biofilms. Surprisingly, despite biofilm stimulation, BM-MSCs maintained strong adhesion properties and spindle-like morphology, particularly on PG-enriched WPI coatings. The metabolic activity of the cells in contact with WPI/PG coatings remained unchanged, indicating good biocompatibility. Furthermore, WPI/PG coatings promoted the expression of key osteogenic markers, facilitating enhanced bone formation and mineralization, especially with 0.5% PG added after autoclaving. Additionally, these coatings significantly reduced the expression of pro-inflammatory markers, highlighting their anti-inflammatory potential. In conclusion, our findings suggest that WPI/PG coatings can not only improve bone–implant integration but also reduce inflammation in biofilm-prone environments, offering an inexpensive and promising strategy for orthopedic and dental implant applications. However, further studies are needed to fully explore their antimicrobial potential.

## Figures and Tables

**Table 1:** WPI coating denominations with and without PG.

Coating Denomination	Description
[WPI] autoclave	WPI fibrillar coating autoclaved
[WPI + PG 0.1%] autoclave	WPI + PG 0.1% mixed coating autoclaved
[WPI + PG 0.5%] autoclave	WPI + PG 0.5% mixed coating autoclaved
[WPI] autoclave + PG 0.1%	WPI fibrillar coating autoclaved + PG 0.1% added after
[WPI] autoclave + PG 0.5%	WPI fibrillar coating autoclaved + PG 0.5% added after

**Table 2:** Primers targeting 16S rRNA used in qPCR analyses.

Bacteria Species	Primer Sequence (5′ to 3′) Forward	Primer Sequence (5′ to 3′) Reverse
*S. mitis*	GATACATAGCCGACCTGAG	CCATTGCCGAAGATTCC
*P. gingivalis*	AGGCAGCTTGCCATACTGCG	ACTGTTAGCAACTACCGATGT
*A. actinomycetemcomitans*	GAACCTTACCTACTCTTGA-CATCCGAA	TGCAGCACCTGTCT-CAAAGC
*F. nucleatum*	GGATTTATTGGGCGTAAAGC	GGCATTCCTACAAATATCTACGAA

**Table 3:** Primers used for RT-qPCR.

Gene	Primer Sequence (5′ to 3′) Forward	Primer Sequence (5′ to 3′) Reverse
glyceraldehyde-3-phosphate dehydrogenase (*GAPDH*)	GAAGGTGAAGGTCGGAGTC	GAGATGGTGATGGGATTTC
RUNX family transcription factor 2 (*RUNX2*)	TCTTAGAACAAATTCTGCCCTTT	TGCTTTGGTCTTGAAATCACA
collagen type I alpha 1 chain (*COL1A1*)	GGTCAAGATGGTCGCCCC	GGAACACCTCGCTCTCCAG
alkaline phosphatase (*ALPL*)	CCTCGTTGACACCTGGAAGAG	TTCCGTGCGGTTCCAGA
osteopontin (*SPP1*)	CGAGGTGATAGTGTGGTTTATGG	GCACCATTCAACTCCTCGCTTTC
bone gamma-carboxyglutamate protein (*BGLAP*)	CTACCTGTATCAATGGCTGGG	GGATTGAGCTCACACACCT
interleukin-1 alpha (*IL1A*)	CGCCAATGACTCAGAGGAAGA	AGGGCGTCATTCAGGATGAA
interleukin-1 beta (*IL1B*)	TTCGAGGCACAAGGCACAA	AAGTCATCCTCATTGCCACTGT
interleukin-8 (*IL8*)	ATGACTTCCAA-GCTGGCCGTGGCT	TCTCAGCCCTCTTCAAAAACTTCT

## Data Availability

The original contributions presented in this study are included in this article. Further inquiries can be directed to the corresponding author.

## Supplementary Materials

The following supporting information can be downloaded at: https://www.mdpi.com/article/10.3390/polym17111514/s1, Figure S1: Scanning Electron Microscopy (SEM) images of WPI fibril coatings containing PG before autoclaving (top row) and after autoclaving (bottom row). Scale bar: 1 micron. WPI: whey protein isolate; PG: phloroglucinol.
