# Supplementary material for: Anti-Inflammatory and Osteogenic Effect of Phloroglucinol-Enriched Whey Protein Isolate Fibrillar Coating on Ti-6Al-4V Alloy
Source: Polymers (Basel). 2025 May 29;17(11):1514. doi: 10.3390/polym17111514 (PMC12157878; doi:10.3390/polym17111514)
Supplement: Supplementary file 1 [file polymers-17-01514-s001.zip › polymers-3497013-supplementary.pdf]

Supplementary Information

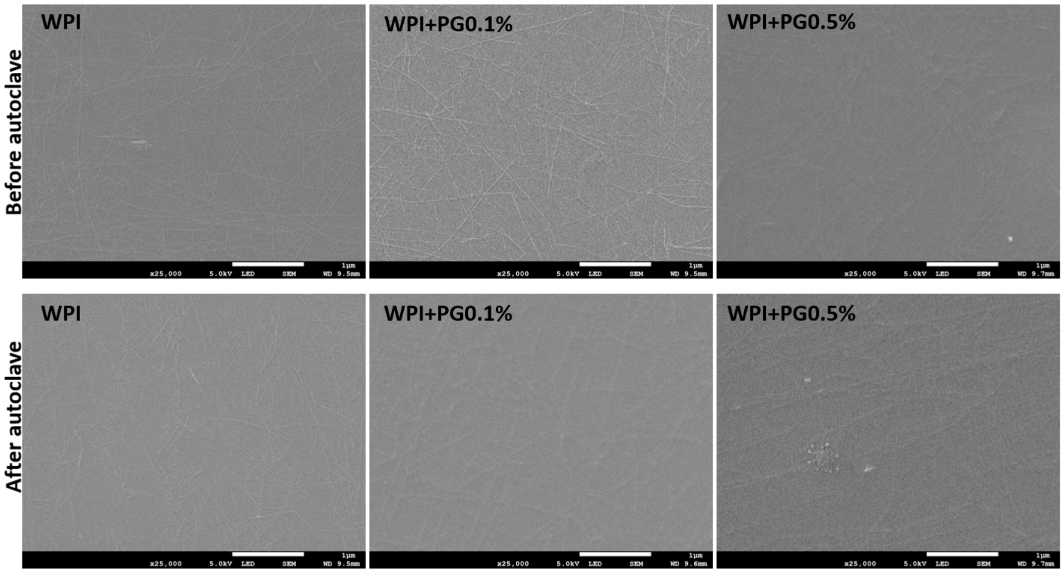

Figure S1. Scanning Electron Microscopy (SEM) images of WPI fibril coatings containing PG before autoclaving (top row) and after autoclaving (bottom row). Scale bar:1 micron. WPI: whey protein isolate; PG: phloroglucinol.
